# The Telomere-to-Telomere Genome of Jaboticaba Reveals the Genetic Basis of Fruit Color and Citric Acid Content

**DOI:** 10.3390/ijms252211951

**Published:** 2024-11-07

**Authors:** Long Zhao, Zixuan Li, Sirong Jiang, Chengcai Xia, Ke Deng, Biao Liu, Zihao Wang, Qi Liu, Miaohua He, Meiling Zou, Zhiqiang Xia

**Affiliations:** School of Breeding and Multiplication (Sanya Institute of Breeding and Multiplication), Hainan University, Sanya 572025, China; longz0322@hainanu.edu.cn (L.Z.); 22210901000059@hainanu.edu.cn (Z.L.); jiangdong2468@163.com (S.J.); 23110901000043@hainanu.edu.cn (C.X.); dengker87@163.com (K.D.); 22220951310081@hainanu.edu.cn (B.L.); 22210901000044@hainanu.edu.cn (Z.W.); missliuliuqiqi@163.com (Q.L.); echohmh@163.com (M.H.)

**Keywords:** *Myrciaria cauliflora*, evolutionary, skin color, citric acid cycle, phosphoenolpyruvate carboxykinase

## Abstract

Jaboticaba is a typical tropical plant that blossoms and bears fruit on the tree trunks and branches. The fruits resemble grapes in appearance and texture and are also known as “treegrapes”. Currently, research on the genomics of jaboticaba is lacking. In this study, we constructed an integrated, telomere-to-telomere (T2T) gap-free reference genome and two nearly complete haploid genomes, thereby providing a high-quality genomic resource. Furthermore, we unveiled the evolutionary history of several species within the Myrtaceae family, highlighting significant expansions in metabolic pathways such as the citric acid cycle, glycolysis/gluconeogenesis, and phenylpropanoid biosynthesis throughout their evolutionary process. Transcriptome analysis of jaboticaba fruits of different colors revealed that the development of fruit skin color in jaboticaba is associated with the phenylpropanoid and flavonoid biosynthesis pathways, with the flavanone 3-hydroxylase (*F3H*) gene potentially regulating fruit skin color. Additionally, by constructing the regulatory pathway of the citric acid cycle, we found that low citric acid content is correlated with high expression levels of genes such as thiamin diphosphate (*ThDP*) and low expression of phosphoenolpyruvate carboxykinase (*PEPCK*), indicating that *PEPCK* positively regulates citric acid content. These T2T genomic resources will accelerate jaboticaba pepper genetic improvement and help to understand jaboticaba genome evolution.

## 1. Introduction

Jaboticaba (*Myrciaria cauliflora*) is an evergreen shrub that originated in South America. Its fruit and flowers both grow on the trunk rather than on the branches. Flowering and fruiting in this plant occur throughout the year, with flowers, unripe fruits, and ripe fruits intermixed on the trunk. Owing to its unique flavor, aroma, color, and bioactive components, the plant exhibits high industrial applicability. The fruits are mostly spherical in shape with a deep purple color, ranging in diameter from 1.6 to 4.0 cm. They consist primarily of an exocarp with a high lipoprotein concentration, a flesh rich in carbohydrates, and one or more high-protein seeds [1,2,3].

Jaboticaba has extremely high added value and is rich in proteins, minerals, and various bioactive compounds, especially anthocyanins, phenolic acids, organic acids, and tannins [4,5,6]. Research indicates that the peel of black jaboticaba accumulates a significant quantity of anthocyanin, surpassing even the anthocyanin content found in blueberry and black raspberry [7,8]. Moreover, the skin of *Myrciaria cauliflora* primarily contains six organic acids (citric acid, malic acid, quinic acid, oxalic acid, shikimic acid, and fumaric acid). Among them, the content of citric acid far exceeds that of the other organic acids, with a concentration of approximately 18.8 ± 0.1 mg/100 g dry weight [4]. Citric acid has found numerous applications in various industrial sectors, functioning as an acidifier or preservative and even enhancing the flavor and aroma of food. Furthermore, it serves as an antioxidant for vitamins and a pH adjuster in the pharmaceutical industry [9]. It has extensive applicability in the food, cosmetics, medical, and pharmaceutical industries [4,10] and holds potential for application in the development of beverages (such as fruit juice, craft beer, and yogurt), jelly, organic vinegar, natural pigments, moisturizing creams, soaps, shampoos, and baked goods [11,12]. It also has significant medicinal value, exhibiting anti-inflammatory, hypoglycemic, hypolipidemic, analgesic, prostate-protective, antimicrobial, noncytotoxic, antioxidant, wound-healing, and sunscreen properties [13,14,15,16,17,18]. The fruit is rich in dietary fiber [19], demonstrating potential prebiotic properties that can promote the growth of the colonic microbiota, thereby improving the intestinal microecology and facilitating lipid, protein, and mineral metabolism [20,21]. Additionally, the fruit contains various minerals, such as calcium, iron, phosphorus, magnesium, and potassium [22], as well as vitamins, such as retinol, thiamine, riboflavin, niacin, and ascorbic acid [23]. In addition to fruit, the leaves of jaboticaba also contain more than 40 phenolic compounds. Among them, ellagic acid, quercetin 3-O-glucoside, gallocatechin, and epigallocatechin were the most prominent in butanol extracts [5]. Additionally, to address previously unresolved sequence gaps, representative gapless or T2T genome assemblies can be generated to decode the long unexplored “final dark matter” in complex plant genomes [24,25]. Jaboticaba has garnered significant attention due to its high nutritional value and potential applications in the food industry, particularly because its fruits are rich in bioactive compounds such as anthocyanins [7]. Currently, research on the jaboticaba genome is still in its infancy, leading to relatively slow progress in the molecular breeding of jaboticaba. A high-quality reference genome is of great importance in studies such as those on crop improvement, effective tracking of genomic variations, important QTL mapping, and the discovery of novel alleles. Resolving the genomes of different types of jaboticaba can not only enhance our understanding of plants that flower and fruit on trunks and branches but also facilitate full utilization of the beneficial genetic resources of jaboticaba.

In this study, we employed various sequencing strategies to perform de novo assembly and annotation of jaboticaba, thereby obtaining the genetic information of jaboticaba. We systematically analyzed the genetic differentiation of jaboticaba and inferred its evolutionary process, thereby clarifying its evolutionary status. Through transcriptome analysis and citric acid content measurement, we aimed to understand the formation and regulatory mechanisms of fruit peel color and citric acid content in jaboticaba.

## 2. Results

### 2.1. A Telomere-to-Telomere Gap-Free Reference Genome for Jaboticaba

**Figure 1:**
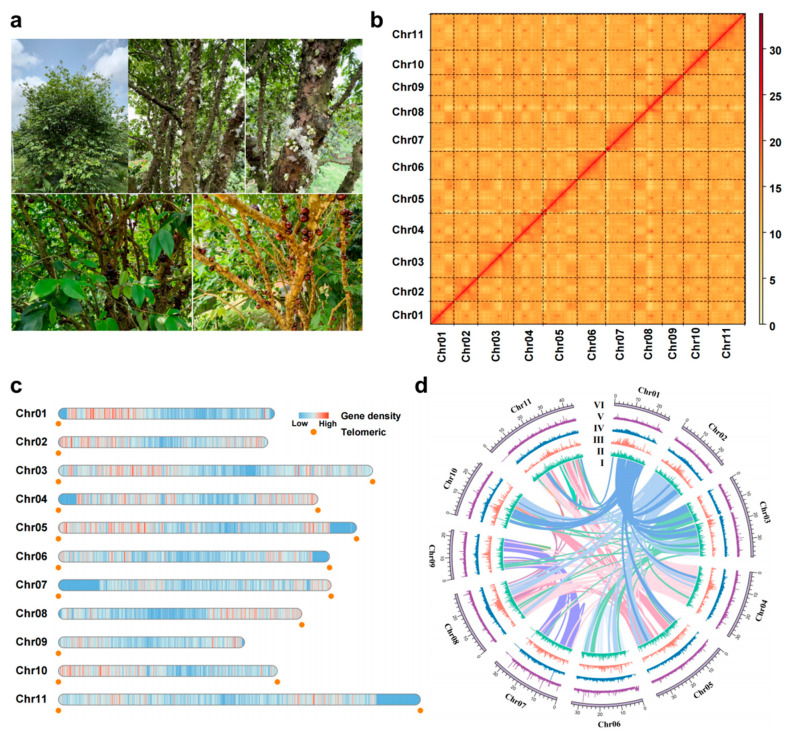
Images, genome assembly, and genomic features of jaboticaba. (**a**) The appearance, trunk, flower, and fruit characteristics of “Evergreen” jaboticaba variety. (**b**) HI-C interaction matrix based on the assembly. (**c**) Gene density and distribution of telomeres in the jaboticaba genome. (**d**) Circle diagram of basic genome information. Concentric circles from inside to outside represent (I) collinearity, (II) LTR–Copia density, (III) LTR–Gypsy density, (IV) LTR density, (V) TE density, and (VI) chromosomes.

The “Evergreen” jaboticaba is currently the most popular jaboticaba variety; it has a short growth cycle, taking only 3 to 4 years to mature and fruit. Its crispy skin and high sweetness make it suitable not only for bonsai cultivation but also for year-round flowering (Figure 1a). The “Evergreen” jaboticaba variety genome was estimated to be ~327 Mb and 340 Mb in length on the basis of flow cytometry and Illumina short reads, respectively (Table S1 and Figure S1a). We also estimated the structure of the jaboticaba genome via the smudge pot method and discovered that “Evergreen” jaboticaba is a typical diploid variety (Figure S1a,b). Different sequencing platforms were employed to construct a high-quality jaboticaba genome. After PacBio HiFi, ONT, next-generation, and Hi-C sequencing, 26.5 Gb (76×), 36.5 Gb (104×), 24.6 Gb (70×), and 55.74 Gb (159×) of raw reads were obtained (Table S2). First, the HiFi, ONT, and Hi-C data were preliminarily assembled via Hifiasm to obtain a genome size of 365.7 Mb with a contig N50 of 33.4 Mb, and then Hi-C technology was applied to generate a chromosome-level genome assembly. NextPolish (v1.4.1) software was combined with next-generation data to correct the genome, and a high-quality and GAP-free jaboticaba genome was ultimately obtained. A total of 351.29 Mb of the assembled genome sequences was anchored to 11 pseudochromosomes (Table 1 and Figure 1b). The lengths of the 11 pseudochromosomes ranged from 22.92 Mb to 44.59 Mb, with an N50 value of 33.4 Mb (Table S3). We used the seven-base telomere sequence (CCCTAAA) as a query sequence to scan the genome and identified 18 telomeres in the 11 chromosomes (Figure 1c).

Multiple methods were used to confirm genome completeness and continuity. First, through the HIC interaction heatmap, the signals of intrachromosomal interactions indicated that all 11 chromosomes were assembled satisfactorily (Figure 1b). The high mapping rates of Illumina short reads (98.66%), HiFi long reads (99.30%), and ultralong reads (96.55%) aligned against the jaboticaba genome suggested the high quality of this assembly (Table 1; Figure S2). Additionally, we assessed the completeness of the long terminal repeat (LTR) sequences, which presented an LTR assembly index (LAI) value of 20.26, meeting the gold standard for genomics (Table 1). Benchmarking universal single-copy ortholog (BUSCO) evaluation of the genome was conducted to verify the integrity of the genome assembly, and the results revealed that 98.9% of the conserved genes were matched, of which 1560 were single-copy homologous genes and 33 were multicopy homologous genes (Table S4).

### 2.2. Genome Annotation and Repeat Sequence Recognition

Homology-based comparisons and structure-based analysis suggested that the annotation of 143.92 Mb transposable elements (TEs) accounted for 40.97% of the jaboticaba genome. The main type of repeat was LTRs, which accounted for 23.05% of the whole genome, including LTR-Copia (5.73%) and LTR-Gypsy (16.54%) (Figure 1d; Table S5). The initially predicted genes were filtered on the basis of gene expression and structure. According to the annotation information, we identified 31,235 protein-coding genes with average gene, coding sequence (CDS), and exon lengths of 2922 bp, 1170 bp, and 206 bp, respectively, and an average exon number of 5.18. BUSCO analysis revealed 97.70% completeness (Table S6).

In addition, we reported the haplotype-resolved diploid “Evergreen” jaboticaba variety genome. Finally, a total of 674.73 Mb of the diploid jaboticaba assembled genome sequence was anchored to 22 chromosomes, with contig and scaffold N50 values of 26.84 Mb and 31.17 Mb, respectively (Table 1 and Table S3; Figure S3). According to the annotation information, there were 62,904 protein-coding genes (Table S6).

### 2.3. Evolution of the Jaboticaba Genome

**Figure 2:**
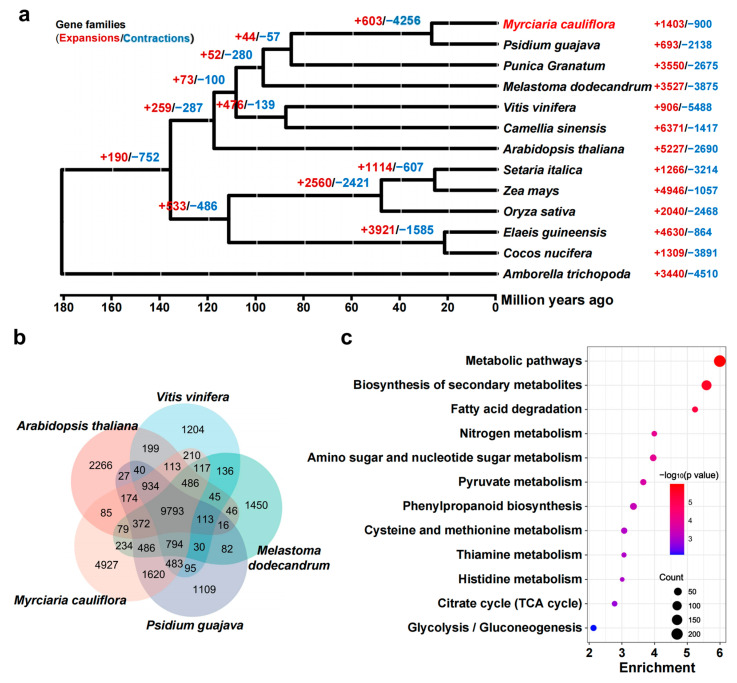
Phylogenetic and comparative genomic analysis of jaboticaba and other species. (**a**) Phylogenetic analysis of 12 species and *Myrciaria cauliflora*. The black characters are the estimated divergence times (in Ma) based on single-copy orthologous group inference. (**b**) Venn diagram of gene family clustering. The numbers represent the number of gene families. (**c**) KEGG enrichment analysis of jaboticaba-specific gene families.

After gene prediction, a phylogenetic tree was constructed on the basis of single-copy orthologous genes clustered. To clarify the phylogenetic relationships of jaboticaba and 12 other species, a phylogenetic tree was constructed to estimate the divergence time. The divergence between Myrtaceae plants in Myrtales and *Melastoma dodecandrum* in Melastomataceae occurred 97 million years ago, whereas the divergence between jaboticaba and the congenerous *Psidium guajava* took place 27 million years ago (Figure 2a). Among them, 1403 genes were found to have undergone expansion, while 900 genes underwent contraction in jaboticaba (Figure 2a). When the gene families of jaboticaba were compared with those of the congenerous guava, as well as *Melastoma dodecandrum*, *Vitis vinifera*, and *Arabidopsis thaliana*, the results suggested that 9793 gene families were shared among these species. In particular, jaboticaba harbored 4927 unique gene families (Figure 2b). Through KEGG enrichment analysis, it was found that the unique gene families in jaboticaba were significantly enriched in several metabolic pathways. Additionally, pathways related to fruit flavor and color, such as the citrate cycle (TCA cycle), glycolysis/gluconeogenesis, and phenylpropanoid biosynthesis, were also identified (Figure 2c).

**Figure 3:**
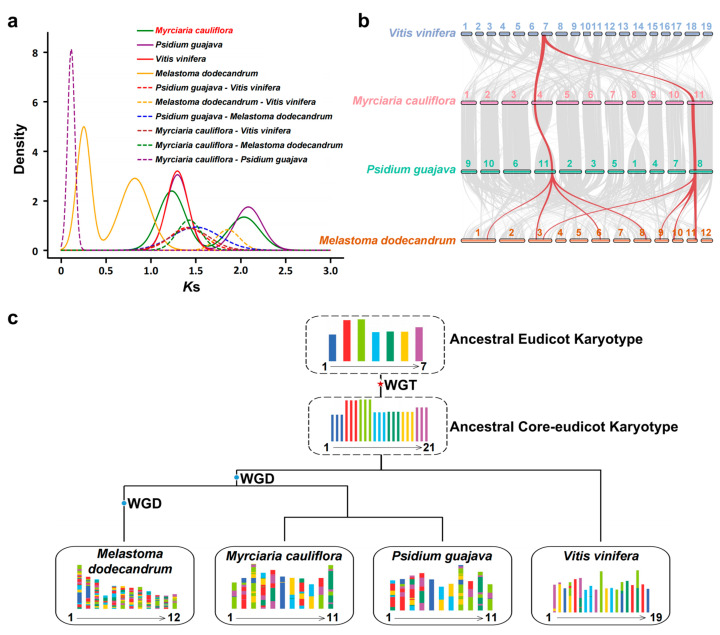
Whole-genome duplication and genome evolution of *Myrciaria cauliflora*. (**a**) Synonymous substitutions per synonymous site (ks) plot of WGD events detected in *Myrciaria cauliflora*. (**b**) Collinearity patterns among *Vitis vinifera*, *Myrciaria cauliflora*, *Psidium guajava*, and *Melastoma dodecandrum*. (**c**) Evolutionary scenario of genome rearrangements from the Eudicot ancestor to *Myrciaria cauliflora* and other sequenced plant genomes.

To further explore whole-genome duplication (WGD) events during the evolution of jaboticaba, we used *Vitis vinifera*, with a well-established history of ancient polyploidization, as a reference. We calculated the Ks (synonymous substitutions per site) values among paralogous gene pairs in *Myrciaria cauliflora*, *Melastoma dodecandrum*, *Psidium guajava*, and *Vitis vinifera* and plotted their distributions (Figure 3a). The distribution of synonymous substitutions among paralogous genes in the jaboticaba genome presented two distinct peaks, one at Ks = 1.23 and the other at Ks = 2.03, which were similar to the two Ks peaks observed in the *Psidium guajava* genome (Ks = 1.29, Ks = 2.08). By comparing the synteny between the *Myrciaria cauliflora* and *Psidium guajava* genomes, we found that the syntenic regions presented a 1:1 relationship (Figure S4a), indicating that jaboticaba and *Psidium guajava* shared two WGD events. In contrast, the *Melastoma dodecandrum* genome presented peaks at Ks = 0.25 and Ks = 0.28. Research has indicated that *Melastoma dodecandrum* has undergone four WGD events, with the oldest event being common to most eudicots, one event unique to Myrtaceae, and the remaining two events specific to *Melastoma dodecandrum* [26]. Through comparative synteny analyses among *Myrciaria cauliflora*, *Psidium guajava*, *Melastoma dodecandrum*, and *Vitis vinifera*, we observed that multiple syntenic regions between *Myrciaria cauliflora* and *Vitis vinifera* displayed a 2:1 relationship (Figure 3b and Figure S4b), suggesting that *Myrciaria cauliflora* underwent an additional duplication event after diverging from Vitis vinifer. Additionally, multiple syntenic regions among *Myrciaria cauliflora*, *Psidium guajava*, and *Melastoma dodecandrum* exhibited a 4:1 relationship (Figure S4c,d), which was consistent with the results of syntenic depth analysis (Figure S5). On the basis of these findings, we speculate that *Myrciaria cauliflora* has undergone two WGD events: the oldest event shared by most eudicots and a second event common to Myrtaceae, without any additional WGD events. To assess the chromosomal evolution history of *Myrciaria cauliflora*, we conducted comparative genomic analyses with *Psidium guajava*, *Melastoma dodecandrum*, and *Vitis vinifera*. We reconstructed seven ancestral chromosomes of eudicots and identified 817 syntenic regions between *Myrciaria cauliflora* and the ancestral chromosomes. Using the reconstructed ancestral karyotypes (AEK, n = 7 and n = 21), we inferred that the 11 chromosomes of *Myrciaria cauliflora* underwent at least 48 chromosomal fission events and 59 chromosomal fusion events (Figure 3c).

### 2.4. Gene Expression Patterns Related to Coloring in Jaboticaba

**Figure 4:**
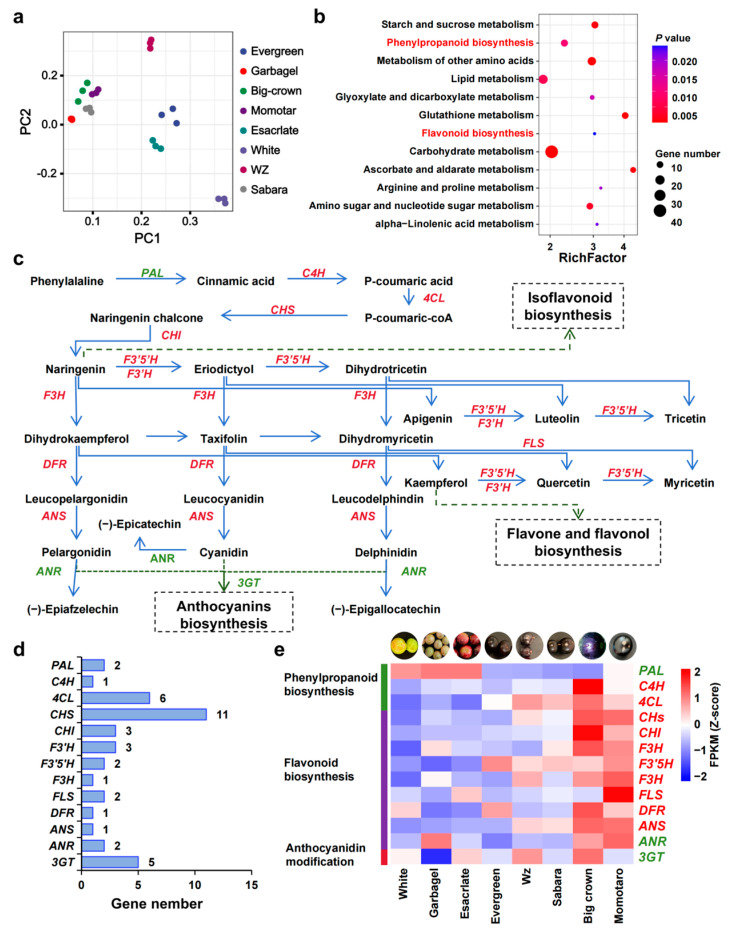
Analysis of anthocyanin biosynthesis-related pathway genes in jaboticaba varieties with different skin colors. (**a**) PCA analysis of the jaboticaba skin expression. (**b**) Enrichment analysis of differential genes between red (Esacrlate, Evergreen, Wz) and white (White, Garbagel)-skinned jaboticoba varieties. (**c**) Analysis of phenylpropanoid biosynthesis, flavonoid biosynthesis, and the anthocyanidin modification pathway in jaboticaba. These pathways were constructed on the basis of the KEGG pathway and the relevant literature. (**d**) Statistical analysis of the number of genes involved in the biosynthesis of anthocyanins. (**e**) Expression analysis of anthocyanin biosynthetic-related genes in jaboticaba varieties with different colors. The heatmaps are drawn according to the FPKM (Z score) values from the transcriptomic datasets.

In plants, flowers, fruits, and vegetative tissues, intricate pigmentation patterns emerge as adaptive responses to both biotic and abiotic challenges [27]. The marked color variation among different varieties of jaboticaba (Figure S6) significantly influences its commercial appeal and consumer preferences. To elucidate the mechanisms underlying the formation and regulation of jaboticaba fruit color, we selected eight jaboticaba fruits with distinct colors and performed transcriptome sequencing on their peels, aligning the data to the “Evergreen” jaboticaba variety genome. Principal component analysis revealed that the three replicates within each sample clustered tightly, whereas substantial differences existed among the samples (Figure 4a). We categorized the eight samples into three groups and conducted differential expression analysis between the red and purple groups and the white group. Notably, the differentially expressed genes (DEGs) between white and red peels were enriched primarily in metabolic pathways such as phenylpropanoid biosynthesis, flavonoid biosynthesis, and starch and sucrose metabolism (Figure 4b). Moreover, the DEGs between white and purple peels were involved mainly in phenylpropanoid biosynthesis, plant hormone signal transduction, and phenylalanine metabolism (Figure S7). Notably, both the phenylpropanoid biosynthesis pathway and the flavonoid biosynthesis pathway are intricately linked to anthocyanin synthesis. We constructed a comprehensive pathway for phenylpropanoid biosynthesis, flavonoid biosynthesis, and anthocyanidin modification in jaboticaba, drawing upon the KEGG pathways and the relevant literature (Figure 4c). Gene copy number analysis within this pathway revealed that *CHS*, *4CL*, and *3GT* were the most abundant, whereas *C4H*, *F3H*, *DFR*, and ANS were present as single copies (Figure 4d). Notably, the expression levels of *C4H*, *4CL*, *CHS*, *CHI*, *F3H*, *F3′5H*, *FLS*, *DFR*, and *ANS* were significantly greater in purple skins (Sabara, Big crown, Momotaro) than in skins of other colors varieties (Figure 4e; Table S7). *F3H* is a single-copy gene whose expression increases with skin color intensity. *F3H* serves as a “pivotal” enzyme at the branching point between anthocyanin and flavonol biosynthesis (Figure 4c), converting (2S)-flavanones to (2R, 3R)-dihydroflavonols such as dihydrokaempferol, taxifolin, and dihydromyricetin [28]. Our findings indicate that *F3H* potentially regulates jaboticaba skin color.

### 2.5. Regulatory Mechanisms of Citric Acid Content in Jaboticaba Variety

**Figure 5:**
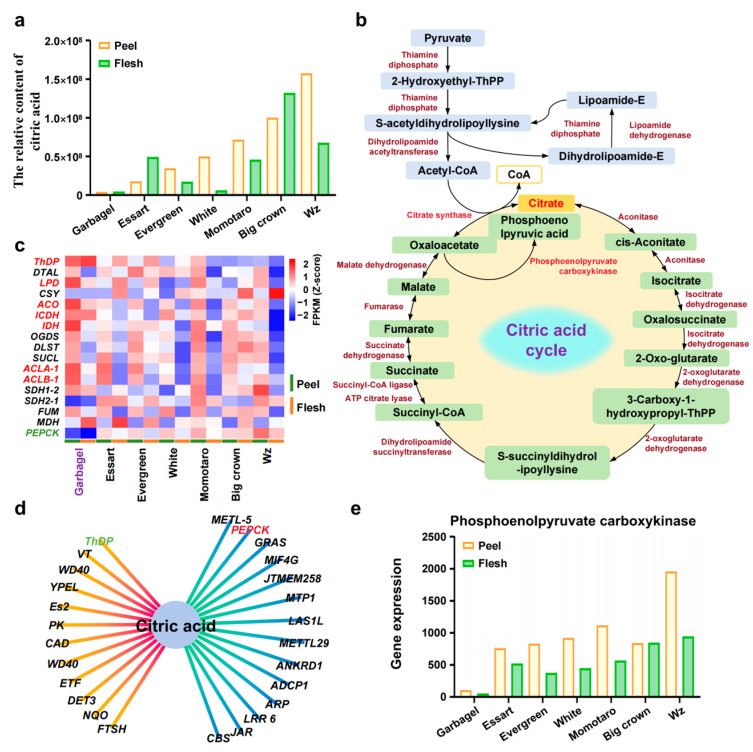
Analysis of the citric acid content and metabolic pathway in jaboticaba. (**a**) Determination of relative citric acid content in jaboticaba flesh and skin. (**b**) Analysis of the citric acid pathway in jaboticaba. (**c**) Heatmap of expression levels of citric acid-related genes in the peel and flesh of different varieties of jaboticoba. (**d**) Analysis of genes related to citric acid content. (**e**) The expression level of the *PEPCK* gene in jaboticaba flesh and skin.

The acidity of fruits, stemming from the presence of organic acids, constitutes a crucial aspect of their sensory quality. Malic acid and citric acid are the primary acids found in most mature fruits. Therefore, understanding the factors influencing the concentration of these acids in fruit cells is vital for enhancing fruit quality [29]. We analyzed the citric acid content in seven jaboticaba varieties with substantial acidity variations and found that the Garbagel variety presented significantly lower citric acid levels in both its skin and pulp than the other jaboticaba varieties did (Figure 5a; Table S8). To elucidate this phenomenon, we constructed a regulatory pathway for the citric acid cycle in jaboticaba on the basis of the KEGG pathway and the relevant literature. Notably, genes such as *ThDP*, *LPD*, *ACO*, *ICDH*, *IDH*, *ACLA-1*, and *ACLB-1* were expressed at significantly higher levels in the low-citric acid Garbagel variety than in the high-citric acid jaboticaba varieties (Figure 5b,c; Table S9). Correlation analysis revealed that the expression of phosphoenolpyruvate carboxykinase (*PEPCK*) in the citric acid cycle was positively correlated with the citric acid content in both the skin and pulp, whereas thiamine diphosphate (*ThDP*) expression was negatively correlated with the citric acid content (Figure 5d; Table S10). Further analysis revealed that *PEPCK* expression was not only proportional to citric acid content but also exceptionally low in the Garbagel jaboticaba variety (Figure 5e), suggesting that *PEPCK* positively regulates citric acid content.

## 3. Discussion

The Myrtaceae family ranks as the eighth largest flowering plant family, encompassing 132 genera and 5950 species, and is predominantly distributed across subtropical and tropical regions of the world [30,31]. Within this family, jaboticaba stands out as a unique evergreen shrub, boasting exceptional industrial applicability owing to its distinctive flavor, aroma, color, and bioactive components [32]. However, the lack of comprehensive genomic analysis of jaboticaba has hindered the advancement of molecular breeding of this plant. This study reports the assembly of a telomere-to-telomere (T2T) genome for jaboticaba. We explored the formation and regulation of fruit skin color and citric acid content, underscoring the importance of these genomic resources for the molecular breeding and genetic improvement of jaboticaba.

Ancient WGD events, also known as ancient polyploidization, are prevalent in plants and represent a potent evolutionary force driving the emergence of novel gene functions and species [33]. To gain insights into the evolutionary trajectory of jaboticaba, we compared its evolutionary relationship with *Psidium guajava* and other peripheral species. Our findings reveal that jaboticaba and *Psidium guajava* share a recent common WGD event, and collinearity analysis further indicated that jaboticaba retains ancestral polyploid features similar to those of other Myrtaceae species, which aligns with previous research [30]. Notably, we identified jaboticaba-specific gene families involved in pathways such as the citrate cycle (TCA cycle), glycolysis/gluconeogenesis, and phenylpropanoid biosynthesis, which are associated with fruit flavor and color, suggesting a connection of these families to the domestication of this unique tropical fruit.

Jaboticaba, an extraordinary tropical plant whose fruit grows on the trunks, exhibits marked color variation among mature fruit (with colors ranging from light green to white, yellow, red, purple, orange, and blue). Our study revealed significant differences in the *F3H* gene among jaboticaba varieties, enabling clear differentiation between red and purple fruit and fruit of other colors. *F3H* serves as a key enzyme in the biosynthetic pathways of flavonols, anthocyanins, and proanthocyanidins [34,35]. Positioned downstream in the flavonoid metabolism pathway, *F3H* catalyzes a branch point that divides flavonoids into anthocyanins and other flavonoid compounds [36]. By modulating *F3H* activity or gene expression, we can influence the catalytic activity and transcriptional levels of flavanones, thereby regulating the flux distribution among different branches of the flavonoid metabolism pathway [37]. Moreover, *F3H* has been shown to alter plant anthocyanin types and contents, increasing flower coloration and ornamental value [38,39]. Additionally, regulating anthocyanin content through *F3H* genes can bolster plant stress adaptability and survival capabilities [40].

The acidity of fruits, resulting from the presence of organic acids, is a crucial component of their sensory quality. Citric acid plays a decisive role in determining the acidity of ripe fruits. Thus, understanding the factors that affect the concentration of these acids in fruit cells is essential for improving fruit quality [29]. This study revealed significant variations in citric acid content among different jaboticaba varieties, which has significant evolutionary implications. This finding aligns with research in citrus fruits, where considerable differences in citric acid content among different citrus varieties lead to distinct sour and non-sour tastes [41]. Additionally, citric acid is a key component of citrus flavor, significantly influencing the process of citrus domestication [42]. Our study revealed that the “Garbagel” jaboticaba variety, which presented the lowest citric acid content, also presented the lowest expression level of the *PEPCK* gene. Notably, the expression level of the *PEPCK* gene is directly proportional to the citric acid content. *PEPCK* serves as a key regulatory enzyme in the gluconeogenesis pathway, catalyzing the conversion of oxaloacetate to phosphoenolpyruvate (*PEP*) [43]. Gluconeogenesis is associated with the release of metabolites from the TCA cycle in vacuoles. For example, reducing malate concentrations can inhibit *PEPCK* activity and decrease *PEP* concentrations, thereby promoting the shift from gluconeogenesis to glycolysis [44]. Furthermore, *PEPCK* may participate in the degradation of citric acid in citrus fruits and the transformation of glycolate in apples and kumquats [45,46,47].

## 4. Materials and Methods

### 4.1. Genome Sequencing

High molecular weight genomic DNAs were extracted from “Evergreen” jaboticaba variety young leaves using the CTAB method and evaluated using a NanoDrop 2000 and Qubit 3.0 Fluorometer. The Nanopore sequencing platform was utilized to sequence the DNA samples. Failed reads were excluded from the raw data, and fragments shorter than 10 kb were filtered using Filtlong v0.2.4 software (https://github.com/rrwick/Filtlong, accessed on 3 November 2024). Additionally, HiFi sequencing was conducted on the PacBio platform, and the quality of the raw data was assessed using CCS v6.0.0 software. Data that were sequenced in fewer than three cycles, low-quality subreads, and data with signal-to-noise ratios below 2.5 were all filtered out. Hi-C technology was employed to explore the spatial positional relationship of chromatin DNA in the genome, and clean data were obtained after removing chimeric sequences and low-quality sequences from the raw data. Next-generation genome sequencing was conducted for subsequent read error correction.

### 4.2. Genome Size Estimation

The genome size was estimated through k-mer frequency analysis. The distribution of k-mers depends on the characteristics of the genome and follows a Poisson distribution. Before assembly, the 19-mer distribution of CCS reads was generated via Jellyfish (v2.2.6) [48]. The smudge plot method was employed to estimate the genome structure of the jaboticaba genome by analyzing heterozygous k-mer pairs [49]. Additionally, a previously reported flow cytometry method was utilized to estimate the genome size of jaboticaba [50].

### 4.3. Genome Assembly

Hifiasm (v0.15.1-r334) [51] was used to perform trio assembly by combining HiFi reads, ONT ultralong reads, and Hi-C reads, resulting in a contig version of the genome. The Pruge_haplotig (v.1.1.0) [52] tool was applied to process genomic heterozygous regions to remove redundancy in the genomes, using default parameters with a few exceptions: -a 50. The pairwise interactions between contigs were subsequently converted into “.hic” files via 3D-DNA and Juicer (v1.6) software, and Juicebox (v2.17.00) software was subsequently utilized for manual ordering and orientation [53,54]. After removing the heterozygous sequences, 100 N bases were inserted to fill the gaps, resulting in the final chromosome-level genome sequence. When comparing gap-filling data and genome gap intervals, gaps were filled at three levels based on the basis of priority: “genome version after error correction > HiFi data > ONT ultralong data”. On the basis of the two haplotype scaffold contigs obtained from Hifiasm assembly, diploid scaffolding and correction were further performed via 3D-DNA and Juicer software. Finally, NextPolish was applied to correct the final assembled genome to ensure the base accuracy of the assembly results [55].

### 4.4. Transposable Element Annotation

For the identification of repeat sequences, we utilized EDTA2 [56], a comprehensive pipeline tool for annotating repeat sequences. We downloaded known SINE/LINE sequences from the SIINE base database (https://sines.eimb.ru/, accessed on 3 November 2024) and submitted them to the curatedlib parameter of EDTA2 (v2.2.0) for initial annotation. We subsequently classified the unclassified LTRs in the initial annotation results. We further classified them via DeepTE [57]. Finally, the EDTA2 pipeline was reused to obtain the final results.

### 4.5. Gene Prediction and Functional Annotation

To predict the protein-coding genes in the genomes, we employed a combined approach integrating homology-based prediction, de novo prediction, and transcriptome-based prediction. For homology-based prediction, we utilized protein sequences from the same family, such as *Psidium guajava* [30], as well as other species, including *Arabidopsis thaliana* [58], *Oryza sativa* [59], *Microcitrus australasica* [60], *Macadamia integrifolia* [61], *Melastoma dodecandrum* [26], *Solanum tuberosum* [62], and *Michelia figo* [63]. For homology-based prediction, BLASTN (v2.16.0) software [64] was initially used for alignment, with the e-value set to 1 × 10^−5^. The aligned sequences were then extended by 2000 bp upstream and downstream, and GeneWise [65] was applied to identify protein structures. To obtain a comprehensive transcriptome sequence, we performed both de novo and genome-guided assemblies of RNA-Seq data via Trinity (v2.8.5) [66]. After merging these two assemblies, we removed contamination through the UniVec database (https://www.ncbi.nlm.nih.gov/tools/vecscreen/univec/, accessed on 3 November 2024) and aligned the cleaned sequences to the genome via PASA [67] to generate integrated transcript sequences. From the PASA results, we extracted ORFs and performed gene prediction, utilizing the complete gene models in conjunction with homologous proteins for hidden Markov model (HMM) training in Augustus [68] and SNAP [69]. For ab initio gene prediction, we also employed Genemark [70]. Initially, intron sites were identified from RNA-Seq data via STAR (2.7.10) [71], and these sites, along with the genome files, were used as inputs for HMM training in Genemark [70]. The gene models obtained from these steps, the genome after masking repetitive sequences, and homology prediction data were then subjected to gene structure annotation via the Maker 2.31.11 (pipeline) [72].

### 4.6. Phylogenetic Analysis of the Genomes

Using OrthoFinder (v2.5.5) [73], single-copy orthologous genes were identified across jaboticaba and 12 other species. The protein sequences of these single-copy orthologous genes were subjected to a concatenated alignment via MUSCLE (v.3.8.31) [74], followed by another round of concatenated alignment. A phylogenetic tree was then constructed via phyML (v.3.3.20190909) [75]. With the calibrated time points obtained from the TimeTree website, the phylogenetic tree was converted into an ultrametric tree via r8s [76]. The CAFE program, with default parameters, was employed to identify expansions and contractions of gene families at each divergence node and species [77].

### 4.7. Synteny and Ks Analyses

BLASTP (with an e-value threshold of 1 × 10^−5^) was employed to compare all protein sequences for the identification of homologous gene pairs. The JCVI (v1.0.4) (https://github.com/tanghaibao/jcvi, accessed on 3 November 2024) was used to detect intraspecific and interspecific collinear regions and generate collinearity Circos plots. WGDI (v0.6.5) [78] was used to calculate the Ks values of collinear homologous gene pairs and to assess karyotype differentiation among chromosomes. To visualize the Ks distribution, we applied kernel density estimation and fitted the Ks distribution curve via a Gaussian mixture model.

### 4.8. Transcriptome Analysis

We collected peels from 8 jaboticaba fruit samples with notable variations in skin color, and flesh and peels from 7 jaboticaba fruit samples with significant differences in acidity. These samples were snap-frozen in liquid nitrogen and subjected to transcriptome sequencing. Each sample was prepared in triplicate, for use in genome assembly, differential analysis of peel color, and expression analysis of genes related to fruit acidity. Total RNA was isolated with a KingFisher Pure RNA Plant Kit (Thermo Fisher, Waltham, CA, USA) and quantified with a NanoDrop 2000 and Qubit 3.0 Fluorometer. The prepared libraries were sequenced on the Illumina HiSeq 2500 platform according to the manufacturer’s recommended protocol. To quantify gene expression in this study, we trimmed RNA-seq reads from different tissues via the Trimmomatic program [79]. Clean reads were then aligned to the “Evergreen” jaboticaba reference genome via HISAT2 software (v2.0.5) [80] to generate Sam files. SAMtools v1.21 [81] was employed to convert the Sam files to Bam files, which were then sorted. Finally, expression levels (FPKM values) were obtained via Stringtie (v2.2.3) software [82]. Differential gene expression between two samples was analyzed via the DESeq2 R package (v1.20.0) [83]. Genes with an adjusted *p*-value < 0.05 identified by DESeq2 were defined as differentially expressed.

### 4.9. Measurement of Citric Acid Content

LC-MS-based metabolomics analysis was conducted to investigate the metabolic profiles. Seven jaboticaba materials with significant differences in fruit acidity were selected, the flesh and skin were separated, the samples were freeze-dried, and 3 biological replicates were performed for each sample. For metabolite detection, the sample was first ground to powder via a grinder. Then, 70% aqueous methanol was added to the powder to extract the metabolites. For metabolome analysis, samples were analyzed using a high-performance liquid chromatography (HPLC)-based targeted method [84,85]. The analytical conditions were as follows: HPLC: column, shim-pack GISS C18 (pore size 1.9 µm, length 2.1 × 100 mm); solvent system, water (0.04% acetic acid): acetonitrile (0.04% acetic acid); gradient program, 0 min, 5%B; 12.0 min, 95%B; 13.2 min, 95%B; 13.3 min, 5%B; 15.0 min, 5%B; flow rate, 0.4 mL/min; temperature, 40 °C; injection volume: 2 µL. The targeted metabolic profiling analysis was performed using scheduled multiple reaction monitoring (MRM) via the LC-ESI-QQQ-MS/MS system (LCMS-8060, SHIMADZU, Kyoto, Japan). The ESI source operation parameters were as follows: nebulizing gas flow, 3 L/min; heating gas flow, 10 L/min; interface temperature, 500 °C; DL temperature, 250 °C; heat block temperature, 400 °C; drying gas flow, 10 L/min. The recorded data were processed using LabSolutions 5.91 software. The final relative citric acid content in the peel and flesh was determined.

## 5. Conclusions

The current study has unveiled a gap-free reference genome spanning from telomere to telomere, along with two nearly complete haploid genomes, furnishing a genomic resource of exceptional quality. Building upon these discoveries, we hypothesize that jaboticaba has undergone two WGD events: the oldest event is shared by the majority of eudicots, while the second event is common within the Myrtaceae family, with no additional WGD events noted. It is noteworthy that the expression levels of *C4H*, *4CL*, *CHS*, *CHI*, *F3H*, *F3′5H*, *FLS*, *DFR*, and *ANS* were significantly elevated in purple-skinned varieties compared to those with other skin colors. Furthermore, the expression of the *PEPCK* gene appears to be positively correlated with citric acid content, potentially suggesting a regulatory role in increasing its levels.

## Figures and Tables

**Table 1:** Evaluation of “Evergreen” jaboticaba variety genome assembly quality.

Assembly	T2T Genome (n = 11)	Diploid Genome (2n = 22)
**Number of chromosomes**	11	22
**Number of gaps**	0	25
**Assembly length (Mb)**	351.29	674.73
**Contig N50 (Mb)**	33.4	26.84
**Scaffold N50 (Mb)**	33.4	31.17
**Illumina read-mapping rate (%)**	98.66	99.6
**HiFi read-mapping rate (%)**	99.30	99.87
**ONT read-mapping rate (%)**	96.55	97.03
**LTR assembly index (LAI)**	20.26	12.99
**Genome completeness (%)**	98.9	98.8
**GC content (%)**	39.48	39.56
**Number of telomeres**	18	3

## Data Availability

The raw sequencing data of PicBio HiFi reads, ONT reads, Hi-C data, whole-genome re-sequencing, RNA-seq data, and genome assemblies have been submitted to the National Genomics Data Center (https://ngdc.cncb.ac.cn/, accessed on 3 November 2024) under accession number PRJCA031828.

## Supplementary Materials

The following supporting information can be downloaded at https://www.mdpi.com/article/10.3390/ijms252211951/s1.
