# Supplementary material for: The Telomere-to-Telomere Genome of Jaboticaba Reveals the Genetic Basis of Fruit Color and Citric Acid Content
Source: Int J Mol Sci. 2024 Nov 7;25(22):11951. doi: 10.3390/ijms252211951 (PMC11593881; doi:10.3390/ijms252211951)
Supplement: Supplementary file 1 [file ijms-25-11951-s001.zip › ijms-3290990-SI.pdf]

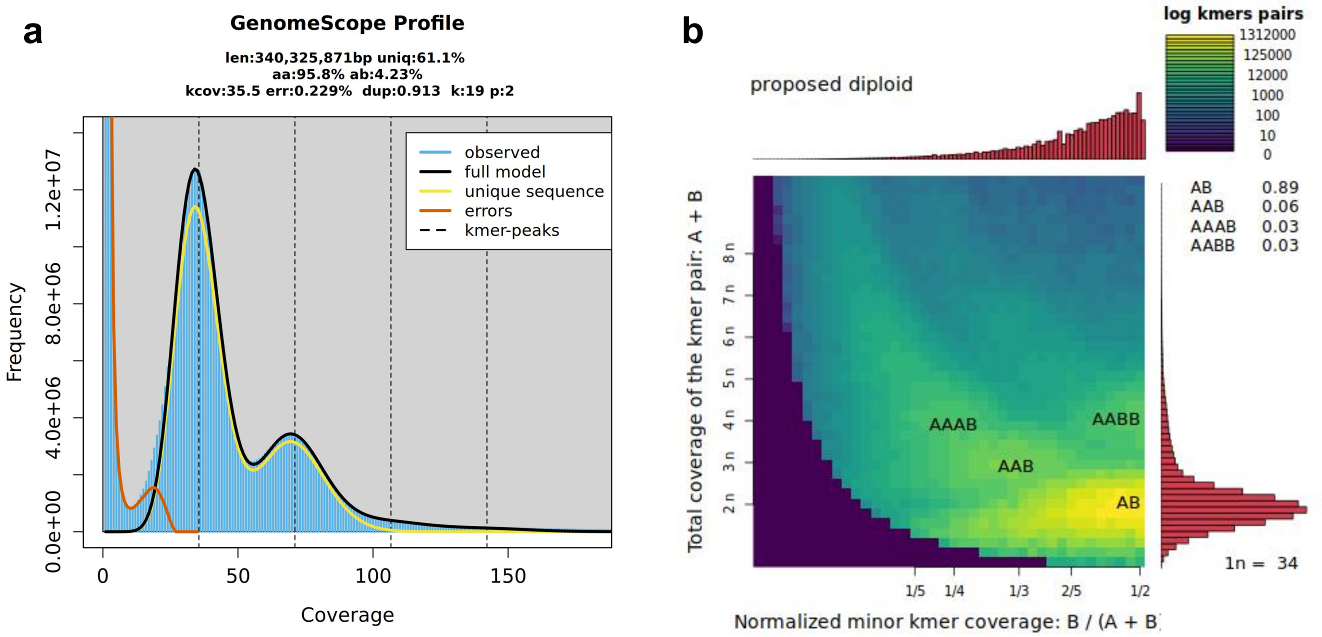

Figure S1. Genomic size and ploidy assessment of "Evergreen" jaboticaba varity. **(a)** Genome survey of "Evergreen" jaboticaba varity genome. (**b)** Estimation of the genome structure of the jaboticaba genome using the Smudgeplot method.

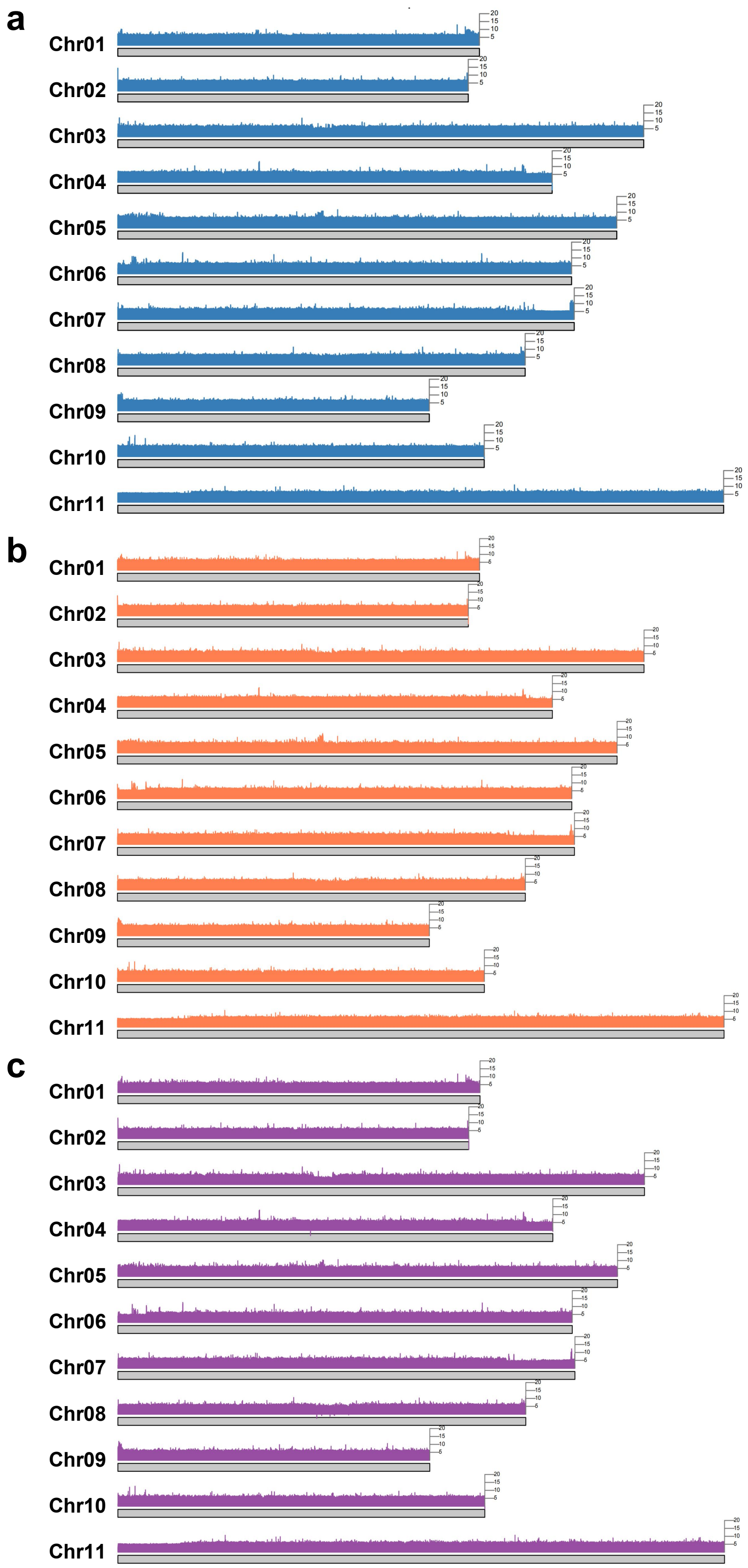

Figure S2. Coverage depth of the final assembly of jaboticaba genome. **(a-c)** Represent Illumina reads, ONT reads, HiFi reads, respectively.

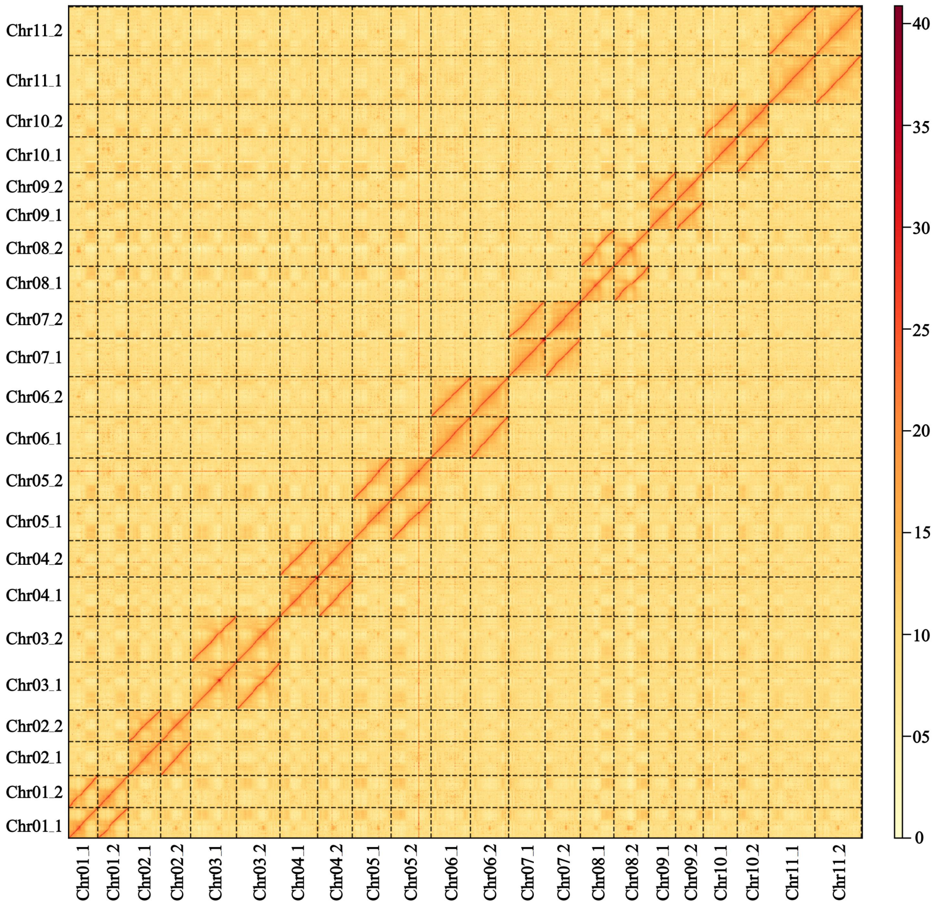

Figure S3. Assessment of the homologous chromosomes assembly based on requencies of intrachromosomal interactions using Hi-C.

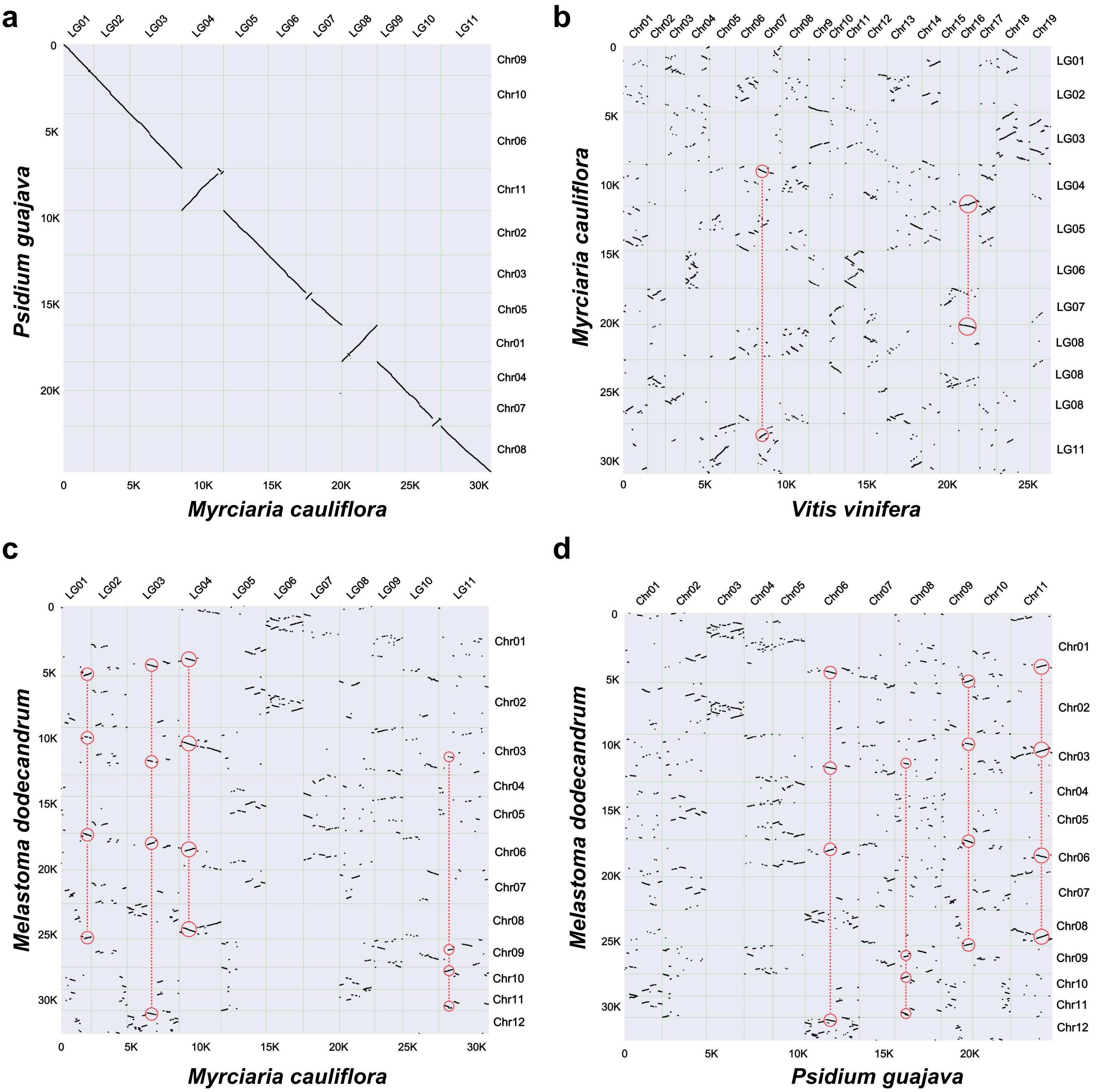

Figure S4. Collinear point diagram of *Myrciaria cauliflora* with orther species. **(a)** Collinear point diagram of *Myrciaria cauliflora* with *Psidium guajava*. **(b)** Collinear point diagram of *Myrciaria cauliflora* with *Vitis vinifera*. The red circle represents a 2:1 relationship in collinear block regions between *Myrciaria cauliflora* and *Vitis vinifera* genome. **(c)** Collinear point diagram of *Myrciaria cauliflora* with *Melastoma dodecandrum*. The red circle represents a 1:4 relationship in collinear block regions between *Myrciaria cauliflora* and *Melastoma dodecandrum* genome. **(d)** Collinear point diagram of *Psidium guajava* with *Melastoma dodecandrum*. The red circle represents a 1:4 relationship in collinear block regions between *Psidium guajava* and *Melastoma dodecandrum* genome.

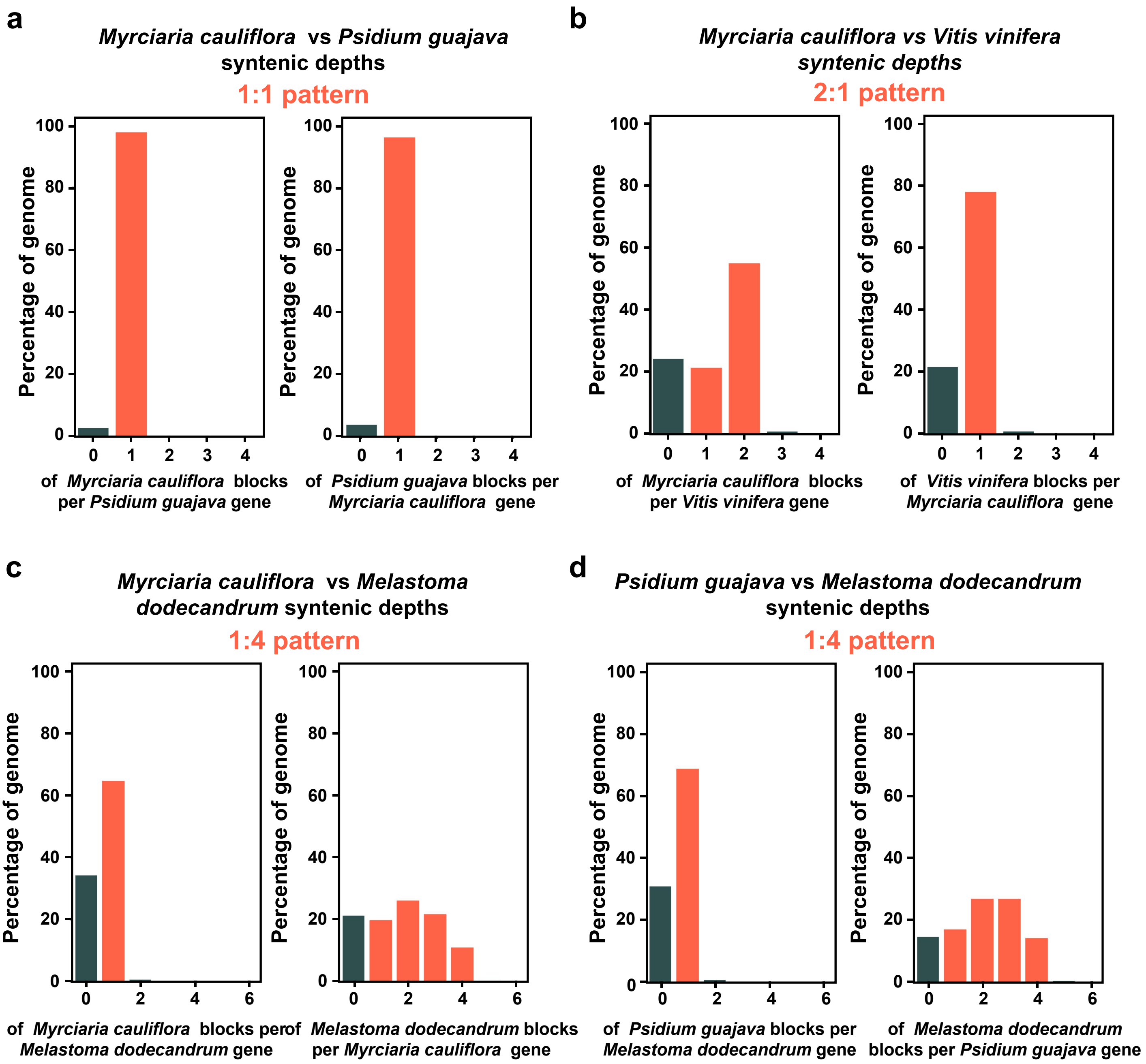

Figure S5. The syntenic depths analysis between *Myrciaria cauliflora* and orther species genomes. **(a)** The syntenic depths analysis between *Myrciaria cauliflora* and *Psidium guajava* genomes. The syntenic depths analysis shows 1:1 pattern between *Myrciaria cauliflora* and *Psidium guajava* genomes. **(b)** The syntenic depths analysis between *Myrciaria cauliflora* and *Vitis vinifera*. The syntenic depths analysis shows 2:1 pattern between *Myrciaria cauliflora* and *Vitis vinifera* genomes. **(c)** The syntenic depths analysis between *Myrciaria cauliflora* and *Melastoma dodecandrum*. The syntenic depths analysis shows 1:4 pattern between *Myrciaria cauliflora* and *Melastoma dodecandrum* genomes. **(d)** The syntenic depths analysis between *Psidium guajava* and *Melastoma dodecandrum*. The syntenic depths analysis shows 1:4 pattern between *Psidium guajava* and *Melastoma dodecandrum* genomes.

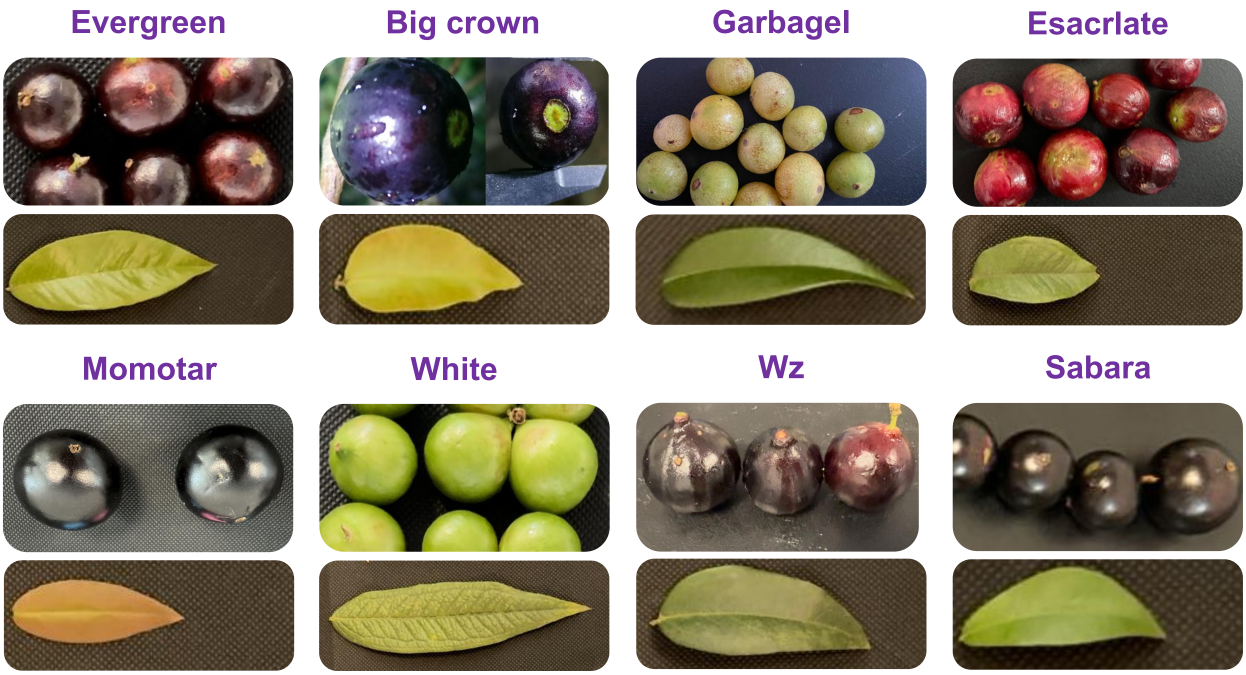

Figure S6. Significant variations in the fruits and leaves of 8 jaboticaba varieties.

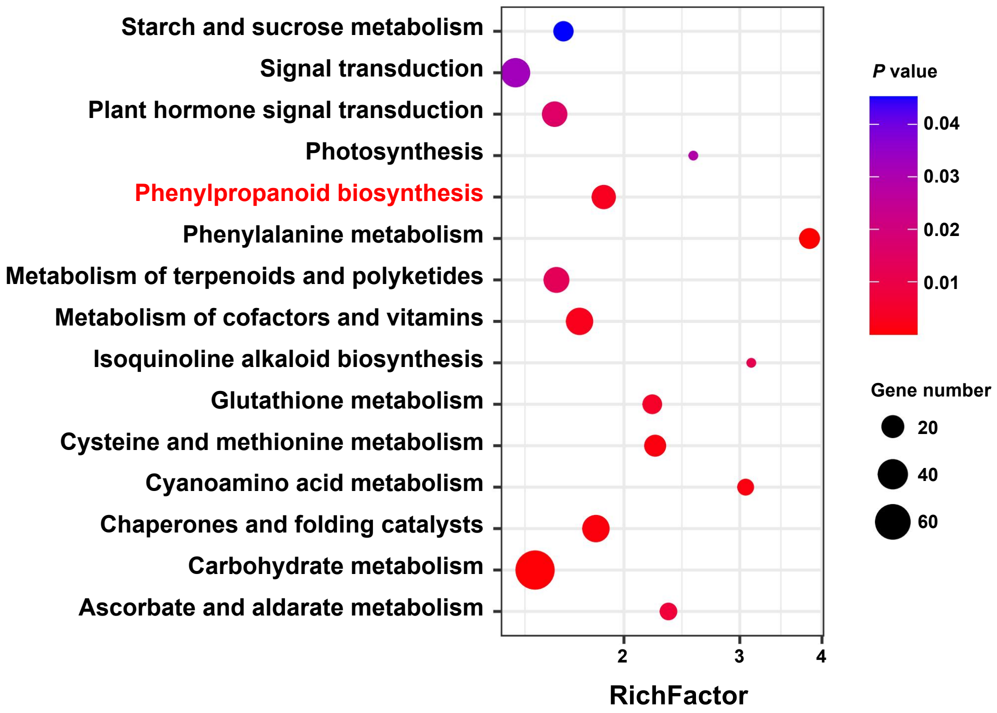

Figure S7. Enrichment analysis of differential genes between purple (Sabara, Big crown, Momotar) and white skinned jaboticaba varieties.

Table S1. Genome size estimation using flow cytometry analysis.

Sample number	Internal reference selection	Internal reference fluorescence intensity	Fluorescence intensity of the sample to be tested	Ratio	Genome size (Mb)
Evergreen 1	Tomato	61.52	21.83	0.35	337
Evergreen 2	Tomato	60.86	20.57	0.34	321
Evergreen 3	Tomato	60.21	20.29	0.34	323

Note: Tomato was used as reference, and three replicates were performed.

Table S2 Summary of the sequence data for jaboticaba genome.

	Raw data (Gbp)	Number of subreads	Depth (X)
**PacBio HiFI**	26.51	1,906,526	75
**Nanopore**	36.48	4,992,986	104
**HiC**	55.74	371,603,568	159
**Illumina**	24.56	231,517,602	70

Note: The assembled genome size (351.29 Mb) was used for sequence depth estimation.

Table S3 General statistics of jaboticaba genome.

chromosome	T2T genome (n=11)	Diploid genome (2n=22)	
		Hap1	Hap2
**Chr01**	26,619,261	24,953,562	25,936,068
**Chr02**	25,795,038	27,609,890	25,468,958
**Chr03**	38,709,171	38,949,123	36,866,863
**Chr04**	31,971,233	32,292,748	29,649,173
**Chr05**	36,727,471	32,768,886	34,032,000
**Chr06**	33,399,941	33,667,101	32,058,509
**Chr07**	33,594,792	31,172,557	30,199,530
**Chr08**	29,984,238	28,389,652	29,704,405
**Chr09**	22,928,200	22,912,040	23,350,511
**Chr10**	26,971,469	28,793,844	26,787,656
**Chr11**	44,591,874	39,587,343	39,291,781
**Total**	351,292,688	674,442,200	

Table S4 BUSCO analysis of jaboticaba completeness.

Description	Genome		Gene	
	Number	Percentage (%)	Number	Percentage (%)
**Complete BUSCOs (C)**	1,593	98.7	1,577	97.7
**Complete and single-copy BUSCOs (S)**	1,560	96.7	1,406	87.1
**Complete and duplicated BUSCOs (D)**	33	2	171	10.6
**Fragmented BUSCOs (F)**	15	0.9	14	0.9
**Missing BUSCOs (M)**	6	0.4	23	1.4
**Total BUSCO groups searched**	1,614	100	1,614	100

Table S5 Statistics of repeat content in the genome.

Class		Number	Length(bp)	% of genome
**LINE**				
	L1	6,603	3,713,628	1.06%
	RTE	1,703	1,117,619	0.32%
**LTR**				
	Copia	29,243	20,115,291	5.73%
	Gypsy	76,006	58,108,755	16.54%
	unknown	1,462	2,733,749	0.78%
**SINE**				
	tRNA	35	5,400	0.00%
	unknown	1,726	304,071	0.09%
**TIR**				
	CACTA	8,768	2,368,027	0.67%
	Mutator	52,780	14,249,208	4.06%
	PIF_Harbinger	17,157	5,090,101	1.45%
	Tc1_Mariner	1,623	419,231	0.12%
	hAT	15,675	6,853,808	1.95%
**nonLTR**				
	pararetrovirus	324	106,105	0.03%
**nonTIR**				
	helitron	31,735	7,994,796	2.28%
**repeat_fragment**		45,351	19,332,128	5.50%
**orthers**		3,820	1,417,612	0.39%
**Total**		294,011	143,929,529	40.97%

Table S6 General statistics of the coding genes for jaboticaba genome.

Accession	T2T genome (n=11)	Diploid genome (2n=22)
No. of genes	31,235	62,904
Annotation completeness (BUSCO, %)	97.70%	98.50%
Repetitive sequences (%)	40.98%	41.55%
Average gene length	2,922	2,897
Average exon length	226	227
Average exon number per gene	5.18	5.13
Average CDS length	1,170	1,103

Table S7. FPKM value of anthocyanin biosynthetic related genes in different colored jaboticaba varieties.

Gene name	White	Garbagel	Esacrlate	Evergreen	Wz	Sabara	Big crown	Momotar
PAL	27.38	29.02	28.94	17.16	16.91	16.06	15.56	21.99
C4H	5.3	26.84	29.62	14.75	34.97	31.24	148.94	44.43
4CL	56.4	104.03	58.97	176.93	258.95	227.9	293.31	216.1
CHS	19.04	149.42	110.39	87.58	266.83	198.1	467.02	434.68
CHI	7.08	41.75	43.07	66.36	85.53	101.78	460.23	237.28
F3'H	3.68	20.79	13.72	12.54	9.84	20.22	33.52	28.73
F3'5'H	23.62	1.28	19.9	129.94	94.49	103.56	98.73	129.03
F3H	24.91	182.62	96.82	78.66	261.44	136.86	286.19	345.84
FLS	3.07	0.18	9.62	1.44	3.09	4.79	3.27	21.97
DFR	96.41	26.28	35.15	116.69	54.56	59.41	145.95	100.14
ANS	1.23	22.75	44.34	46.38	217.63	205.2	362.15	353.66
ANR	5.83	18.12	7.35	2.47	6.3	5.68	16.03	19.64
3GT	6.13	0.22	7.06	4.96	8.67	4.81	9.61	4.94

Table S8. Determination of relative citric acid content in 7 jaboticaba varieties flash and skin.

Name	Peel	Flesh
**Garbagel**	2.19E+06	2.84E+06
**Essart**	1.82E+07	5.02E+07
**Evergreen**	3.55E+07	1.77E+07
**White**	5.08E+07	6.36E+06
**Momotaro**	7.29E+07	4.67E+07
**Big crown**	1.02E+08	1.34E+08
**Wz**	1.60E+08	6.88E+07

Table S9. The FPKM value of PCK gene in 7 jaboticaba varieties flash and skin.

Gene name	Garbagel		Essart		Evergreen		White		Momotaro		Big crown		Wz	
	Peel	Flash	Peel	Flash	Peel	Flash	Peel	Flash	Peel	Flash	Peel	Flash	Peel	Flash
**Thiamine diphosphate (*ThDP*)**	307.4	334.5	191.5	251.9	182.5	259.8	158.2	200.6	240	147.1	145.4	154.7	157.6	129.7
**Dihydrolipoamide acetyltransferase (*DTAL*)**	169.9	86.2	161.6	127.8	213.6	153.4	174.2	152.7	187.3	70.3	135.9	128.5	179.1	64.4
**Lipoamide dehydrogenase (*LPD*)**	231.6	182.8	163.5	208.3	155.7	191.9	130.2	144.3	209.2	160.5	141.8	174.7	195.4	131.6
**Citrate synthase (*CSY*)**	237.1	233.4	237.6	450.7	231.6	319	192.4	380.2	338.8	266.4	271	515.6	276.7	670.7
**Aconitase (*ACO*)**	339.8	185.9	169.1	103.9	199	87	177.9	85	249.1	65.5	188.8	132.6	184.8	71.9
**Isocitrate dehydrogenase 1 (*ICDH*)**	563.5	554.8	355.9	281.5	347.8	247	516.6	322.4	431.1	228.9	401.1	483.5	424.5	156.8
**Isocitrate dehydrogenase (*IDH*)**	254.2	172.9	187.3	167.6	194.9	193.4	174.8	133.9	226.8	180.6	207.1	172.8	164.8	114.7
**2-oxoglutarate dehydrogenase (*OGDS*)**	76	32.8	54	25.4	70	37.8	48.8	22.8	89.1	13.1	67.6	60.6	52.2	13.2
**Dihydrolipoamide succinyltransferase (*DLST*)**	141.6	80.4	117.6	94.2	148.3	107.1	100.5	63.3	154.6	72.5	138.2	159.1	138.4	70.4
**Succinyl-CoA ligase (*SUCL*)**	150.5	90.6	162.3	83.6	139.7	122.5	133	66.6	146.4	84.7	153.5	137.3	141.5	63.8
**ATP-citrate lyase A (*ACLA-1*)**	525	200.6	444	84.2	397.9	138.6	185.3	78.7	298.5	97.9	300	159.8	173.6	82.3
**ATP-citrate lyase B (*ACLB-1*)**	243.3	63.7	198	33	138.1	42.4	101.9	28.4	167.4	29.9	108.9	70.9	129.3	52.5
**Succinate dehydrogenase 1-2 (*SDH1-2*)**	95.2	87.2	62.6	77.3	63	68.1	51.4	76	99.4	53	80.2	84.6	112.4	57.8
**Succinate dehydrogenase 2-1 (*SDH2-1*)**	43.6	52.6	154.3	148.7	91.5	137.4	90.8	80.1	164.7	124.6	73.5	65.2	156.1	124.1
**Fumarase (*FUM*)**	22.5	12.7	20	7.3	19.5	10	24.1	7.6	21.8	9.2	18.2	20.1	19.1	7.1
**Malate dehydrogenase (*MDH*)**	547.6	843.7	507.8	892.3	450.2	741.7	444.7	607.6	632.3	597	427.2	691.3	460.8	574.3
**Phosphoenolpyruvate carboxykinase (*PCK*)**	108.5	56.1	756.9	518.9	826.2	376.6	916.1	438.4	1111.3	556.9	810.2	841	1942	938.7

Table S10. Analysis of significant genes related to citric acid content.

Omics_1	Omics_2	Correlation	P-Value	Description
**Citric**	Jab.sjzs01G000471	0.788046	0.002331	*PEPCK*
**Citric**	Jab.sjzs11G000736	0.793297	0.002076	*ADCP1*
**Citric**	Jab.sjzs09G001996	0.88003	0.000159	*ANKRD1*
**Citric**	Jab.sjzs05G002025	-0.888872	0.00011	*CAD*
**Citric**	Jab.sjzs11G001271	0.890386	0.000103	*CBS*
**Citric**	Jab.sjzs01G001604	-0.806411	0.001532	*CCDC*
**Citric**	Jab.sjzs10G001231	-0.874391	0.000199	*DET3*
**Citric**	Jab.sjzs02G002252	0.782502	0.002626	*DRM1/ARP*
**Citric**	Jab.sjzs04G002539	-0.850007	0.000462	*Es2*
**Citric**	Jab.sjzs11G000339	-0.797926	0.001869	*ETF*
**Citric**	Jab.sjzs05G003169	-0.837306	0.000679	*FTSH*
**Citric**	Jab.sjzs07G001026	0.884019	0.000136	*GRAS*
**Citric**	Jab.sjzs03G001109	0.819927	0.001093	*JAR*
**Citric**	Jab.sjzs02G000622	0.889139	0.000109	*LAS1L*
**Citric**	Jab.sjzs10G001853	0.825899	0.000933	*LRR_6*
**Citric**	Jab.sjzs08G000778	0.828803	0.000862	*METL-5*
**Citric**	Jab.sjzs08G000628	0.797235	0.001899	*METTL29*
**Citric**	Jab.sjzs08G000779	0.807416	0.001495	*MIF4G*
**Citric**	Jab.sjzs09G001251	0.79266	0.002106	*MTP1*
**Citric**	Jab.sjzs08G000009	-0.82637	0.000921	*NCS*
**Citric**	Jab.sjzs10G002432	-0.822379	0.001025	*NQO*
**Citric**	Jab.sjzs02G001818	-0.887113	0.000119	*PK*
**Citric**	Jab.sjzs05G002380	-0.800882	0.001746	*ThDP*
**Citric**	Jab.sjzs06G002322	0.845898	0.000525	*TMEM258*
**Citric**	Jab.sjzs04G000414	-0.808586	0.001453	*WD40*
**Citric**	Jab.sjzs08G000064	-0.868144	0.000251	*WD40*
**Citric**	Jab.sjzs07G000691	-0.816242	0.001201	*YPEL*
**Citric**	Jab.sjzs03G003250	-0.861242	0.000319	*VT*
